# Multiple job holding, working hours, and hypertension by race/ethnicity and sex

**DOI:** 10.1371/journal.pone.0300455

**Published:** 2024-05-21

**Authors:** Caryn N. Bell, Carlos D. Tavares, Jessica L. Owens-Young

**Affiliations:** 1 Department of Social, Behavioral and Population Science, Tulane University School of Public Health and Tropical Medicine, New Orleans, LA, United States of America; 2 Department of Anthropology and Sociology, Lafayette College, Easton, PA, United States of America; 3 Department of Health Studies, American University, Washington, DC, United States of America; Dartmouth Health, UNITED STATES

## Abstract

The number of Americans with multiple jobs is increasing and multiple jobholders work more hours per week. However, the associations between multiple jobholding and hypertension are unknown. The aim of this study was to examine the associations of multiple jobholding with hypertension and determine whether weekly working hours moderated this association. Data from the 2015 National Health Interview Survey on adults (age ≥18 years) were used and included participants who self-identified as non-Hispanic Asian, non-Hispanic Black, Hispanic, or non-Hispanic White in the U.S. (n = 16,926), The associations of multiple jobholding with self-reported hypertension by sex were assessed using modified Poisson regressions. Both the number of working hours per week and race/ethnicity were assessed as moderators using multiplicative interaction terms. Multiple jobholding was not associated with hypertension among women. However, there was a significant three-way interaction such that multiple jobholding was associated with hypertension among non-Hispanic Black men who worked ≥55 hours per week (relative risk = 1.02, 95% confidence interval = 1.01–1.05). The results suggest that the associations between multiple jobholding, number of working hours, and hypertension should be examined at the intersection of race/ethnicity and sex. Future studies should further characterize multiple jobholding and hypertension among non-Hispanic Black men.

## Introduction

The number of Americans who hold multiple jobs has increased in recent years [1,2]. While definitions, measurement, and names for multiple jobholding vary over time and discipline [1,3,4], multiple jobholding can broadly be understood as having two or more jobs at once. The U.S. Census Bureau finds that 7.8% of working Americans were multiple jobholders in 2018 [1,2]. Studies show that adults hold multiple jobs for several reasons including financial need and career advancement [3–5]. There are also demographic differences in the number of multiple jobholders and in temporal changes in multiple jobholding [2,4,6]. For example, 9.1% of women were multiple jobholders compared to 6.6% of men in 2018 and the recent increase in the rate of Americans who hold multiple jobs was only observed among women [2]. There are also race/ethnic differences in multiple jobholding [4,6]. The highest rates of multiple jobholding are found among Black Americans followed by White Americans [4,6]. Asian and Hispanic Americans have comparably lower rates of multiple jobholding [4,6].

Though only a few previous studies have examined the association between multiple jobholding and health, the results are mixed [3,7–16]. For example, a 2020 review found that multiple jobholding is associated with worse psychological well-being, less physical activity, poorer sleep outcomes, and more physical injury [3]. However, Jamal, Baba, & Riviere (1998) study found that multiple jobholders report less stress than those with only one job. Moreover, a 2020 study of Chinese agricultural workers found that having 2 concurrent jobs was associated with better self-rated health, but having three or more jobs was associated with worse self-rated health [7].

It is possible that multiple jobholding is associated with odds of hypertension. Role Theory [17–19] indicates that social roles, such as employment, significantly shape health [20–22]. Specifically, researchers generally finds that employment (vs. non-employment) is associated with better health [23–25]. Some scholars posit that multiple social roles are health protective because connecting to key social institutions (e.g., employment) increases access to important resources such as social support and financial compensation [26,27]. Important for our study, multiple jobholding may result in lower hypertension odds through more robust social networks, social support, and increase access to financial resources. Other role theory researchers suggest multiple social roles result in suboptimal health outcomes through role strain, overload, and conflict [20]. For example, a large literature on the role of work in hypertension has centered on work-related stress and job strain [28,29]. Studies on multiple jobholding apply the concept of role strain suggesting that multiple jobholding is associated with increased levels of stress [30,31]. Studies have also shown that longer working hours are associated with hypertension [32–35]. Multiple jobholders tend to work longer hours per week than those who have only one job [3,4,30,36]. Thus, it is also plausible that working longer hours may lead to role overload, conflict, and strain leaving multiple jobholders with less time to adequately fulfill other roles. Given the limited research on multiple jobholding and hypertension, it is important to explore how insights from Role Theory apply to this association.

The aim of this study is to examine the association between multiple jobholding and the odds of hypertension among adults in the U.S. and whether working hours moderate this association. Given demographic differences in the rates and experiences of multiple jobholding [2–4,6], differences in these associations by race/ethnicity and sex will also be assessed. The results of this study will be used to inform understandings of the health impacts of multiple jobholding across race/ethnicity and sex in the U.S.

## Methods

### Data

Conducted annually by the U.S. Census Bureau for the National Center for Health Statistics, the National Health Interview Survey (NHIS) is a cross-sectional survey of the non-institutionalized civilian population in the U.S. [37,38]. NHIS data is nationally representative and uses a multistage sampling design and includes an oversampling of Black/African Americans, Hispanics, Asians, and older adults. NHIS data are publicly available and fully de-identified. Therefore, the study was exempt with regard to requirements of the Institutional Review Board. NHIS data includes information on participants’ health status, behaviors, healthcare utilization, and other demographics. This study was conducted according to the guidelines laid down in the Declaration of Helsinki and all procedures involving research study participants were approved by the National Center for Health Statistics. The data are de-identified and publicly available. Written informed consent was obtained from all subjects/patients. In 2010 and 2015, a module on occupational health was included that asks several questions about participants’ workplace characteristics [38]. Data from 2015 is the most NHIS dataset in which multiple jobholding is assessed. A random sample of one adult in each household surveyed included 33,672 adults in 2015. The sample for this study included adults (aged ≥18 years) included in the sample adult file whose race/ethnicity was identified as non-Hispanic Asian, non-Hispanic Black, Hispanic, or non-Hispanic White (n = 32,791). After excluding participants with missing data on any analytical variable, a final sample of 17,190 individuals was included in the study.

### Variables

The dependent variable is self-reported hypertension. Participants were asked whether they had ever been told by a doctor that they had hypertension. A dichotomous variable was created where those who responded that they had never been diagnosed with hypertension were given a value of “0” and those who responded that they had been diagnosed with hypertension were given a value of “1”. The primary independent variable was multiple jobholding. Current employment status was obtained by asking participants “Which of the following were you doing last week: working for pay at a job or business, with a job or business but not at work, looking for work, working (but not for pay) at a family-owned job or business, or not working at a job or business and not looking for work”. Those who were working last week, who were with a job or business but not at work last week, or who were working but not for pay at a family-owned job or business were additionally asked about multiple jobholding. Participants were asked if they have more than one job. A dichotomous variable to represent multiple jobholding was created such that those who responded that they did not have more than one job were given a value of “0” and those responded that they did have more than one job were given a value of “1”. The moderating variable was working hours. Participants were asked how many hours they worked last week at all jobs or businesses. A continuous variable to represent weekly working hours was included in regression analyses.

Several demographic and health-related variables were included as covariates. Age was measured as a continuous variable. Marital status was categorized as follows: currently, formerly, or never married. Respondents were asked about their highest level of education. A categorical variable was created, and categories included: not a high school graduate, high school graduate or GED equivalent, Associate’s degree or some college, and Bachelor’s degree or more. Household income was calculated in terms of the percentage of the federal poverty line (FPL). A categorical variable was created with the following categories: <100% FPL, 100–200% FPL, 200–400% FPL, ≥400% FPL. Participants were asked if they had their blood pressure checked by a doctor, nurse, or other health professional in the last 12 months. Those who indicated that they had were given a value of “1” and those who had not were given a value of “0”. Body mass index (BMI) was calculated based on self-reported height and weight. BMI was included as a continuous variable. Variables to indicate respondent who were current drinkers and current smokers were created from data that indicated whether the respondent consumed alcohol currently and currently smoked cigarettes every day or some days. Respondents were considered physically inactive if they did not report participating in any moderate nor vigorous physical activity.

### Statistical analyses

Following the procedure recommended by the National Center for Health Statistics, all analyses used Taylor-linearization procedures for the complex multistage sampling design. All statistical analyses were conducted in STATA Version 17 (StataCorp LP, College Station, TX). Students *t* and chi-square tests were used to assess differences in analytical variables by race/ethnicity and sex. Adjusting for covariates, the associations of multiple jobholding, working hours, and race/ethnicity with hypertension was assessed within sex groups using the *svy subpop* command. Modified Poisson regressions were used because the prevalence of hypertension was not rare (about 31% overall) [39]. Multiplicative interaction terms were used to determine whether the associations between multiple jobholding and hypertension were moderated by longer working hours and race/ethnicity. Associations were considered significant when the p-value was ≤0.05. If a significant three-way interaction between race/ethnicity, multiple jobholding, and working hours was detected, predicted probabilities of hypertension by multiple jobholding, working hours, and race/ethnicity. The *margins* command was used to obtain predicted probabilities of hypertension by multiple jobholding, working hours, and race/ethnicity.

## Results

Table 1 displays descriptive statistics for all analytical variables by race/ethnicity and sex. There were differences in age, distribution across marital status categories, household income, educational attainment, BMI, smoking, drinking, and physical inactivity. There was also racial/ethnic and sex variation in the proportion of participants who held more than one job (p<0.001). For example, 4.4% of Hispanic women reported having multiple jobs while 9.4% of non-Hispanic Black women were multiple jobholders. Number of working hours per week also varied by race/ethnicity and sex (p<0.001). The mean number of weekly working hours among Hispanic women was 35.5 hours compared to 42.1 hours among non-Hispanic White men. There were race/ethnicity and sex differences in hypertension as well (p<0.001). Among Hispanic men, 22.4% reported being diagnosed with hypertension. However, about 41.0% of non-Hispanic Black women reported having hypertension.

**Table 1:** Descriptive statistics of analytical variables by race/ethnicity and sex, National Health Interview Survey 2015.

	Non-Hispanic Asian		Non-Hispanic Black		Hispanic		Non-Hispanic White		
	Women	Men	Women	Men	Women	Men	Women	Men	p-value
	N = 461	N = 564	N = 1,223	N = 828	N = 1,455	N = 1,574	N = 5,374	N = 5,447	
Age (years), mean ± s.d.	45.8 ± 16.2	44.0 ± 16.8	45.3 ± 19.8	44.1 ± 16.8	41.8 ± 18.1	40.4 ± 15.4	50.1 ± 18.5	48.6 ± 17.1	<0.001
Marital status, %									
Currently	71.5	67.6	35.0	47.4	61.6	57.2	61.7	65.9	<0.001
Formerly	13.7	5.7	27.1	16.9	8.8	18.3	23.6	12.9	
Never	14.9	26.7	38.0	35.7	29.6	24.5	14.6	21.2	
Household income (percentage of the federal poverty line), %									
<100%	12.9	10.8	25.5	17.0	26.1	17.2	9.1	7.5	<0.001
100–199%	13.3	17.9	25.1	23.4	29.2	29.5	16.9	14.0	
200–399%	29.8	23.8	28.2	30.8	28.1	31.5	29.2	28.4	
≥400%	44.0	47.5	21.2	28.9	16.6	21.8	44.9	50.2	
Educational attainment, %									
Not a high school graduate	9.6	9.0	13.9	14.3	31.4	32.5	7.5	8.4	<0.001
High school grade/GED equivalent	14.0	13.8	24.8	33.0	27.0	29.5	23.9	24.9	
Some college/ Associate’s degree	20.8	20.2	38.5	31.8	26.8	24.9	33.1	31.2	
≥Bachelor’s degree	55.6	57.0	22.8	20.9	14.9	13.1	35.6	35.5	
Annual blood pressure check, %	82.9	72.1	89.2	78.0	77.3	61.4	90.6	81.6	<0.001
Body mass index (kg/m^2^), mean ± s.d.	26.5 ± 14.6	26.8 ± 11.5	34.0 ± 19.5	30.1 ± 12.6	32.0 ± 18.8	29.8 ± 10.5	31.4 ± 18.1	29.3 ± 9.6	0.031
Current smoker, %	2.6	12.0	13.3	20.9	7.1	13.1	16.0	17.2	<0.001
Current drinker, %	38.3	61.4	52.2	59.7	49.7	68.4	66.2	73.2	<0.001
Physically inactive, %	25.3	23.5	42.7	30.6	36.8	36.9	28.7	26.3	<0.001
Multiple jobholder, %	6.9	8.0	9.1	9.0	4.4	6.3	9.0	8.8	<0.001
Hours worked last week, mean ± s.d.	37.3 ± 12.1	41.2 ± 11.3	37.7 ± 13.5	40.9 ± 12.0	35.5 ± 12.5	41.1 ± 11.3	37.0 ± 13.1	42.1 ± 12.7	<0.001
Self-reported hypertension, %	23.4	23.7	41.0	37.3	23.4	22.5	30.4	34.4	<0.001

Notes: s.d. = standard deviation.

The associations of multiple jobholding, working hours, and race/ethnicity with relative risk of hypertension are shown in Table 2 by sex. Among women, neither multiple jobholding nor working hours were associated with hypertension in Model 1. However, non-Hispanic Black women had 80% higher relative risk of hypertension compared to non-Hispanic White women (relative risk (RR) = 1.80, 95% confidence interval (CI) = 1.60–2.03). In Model 2, there were no significant interactions between multiple jobholding, working hours, and race/ethnicity on hypertension. There were no associations between multiple jobholding and working hours among men in Model 1. However, non-Hispanic Black men had 21% higher relative risk of hypertension compared to non-Hispanic White men (RR = 1.21, 95% CI = 1.05–1.38), while Hispanic men had 15% lower relative risk of hypertension (RR = 0.85, 95% CI = 0.73–0.99). In Model 2, there was a significant three-way interaction between multiple jobholding, working hours, and race/ethnicity specifically comparing non-Hispanic Black men to non-Hispanic White men (RR = 1.02, 95% CI = 1.01–1.05).

**Table 2:** Associations between multiple jobholding, working hours, race/ethnicity, and hypertension by sex, National Health Interview Survey 2015.

	Women		Men	
	N = 8,513		N = 8,413	
	Model 1	Model 2	Model 1	Model 2
	RR (95% CI)	RR (95% CI)	RR (95% CI)	RR (95% CI)
Multiple jobholder	1.02 (0.85–1.24)	0.69 (0.32–1.46)	1.07 (0.91–1.27)	1.17 (0.65–2.09)
Number of hours worked	1.00 (0.99–1.01)	1.00 (0.99–1.01)	1.00 (0.99–1.01)	1.00 (0.99–1.01)
Race/ethnicity				
Non-Hispanic White	1.00	1.00	1.00	1.00
Non-Hispanic Asian	1.23 (0.94–1.60)	2.25 (1.07–4.69)	0.98 (0.80–1.19)	2.10 (1.07–4.13)
Non-Hispanic Black	1.74 (1.55–1.96)	1.51 (1.06–2.16)	1.18 (1.03–1.35)	1.78 (1.16–2.71)
Hispanic	0.98 (0.85–1.14)	0.94 (0.54–1.63)	0.93 (0.80–1.08)	1.45 (0.93–2.24)
Multiple jobholder × Number of hours worked		1.01 (0.99–1.02)		0.99 (0.98–1.01)
Multiple jobholder × Race/ethnicity				
Non-Hispanic White		1.00		1.00
Non-Hispanic Asian		2.75 (0.14–54.22)		7.61 (0.93–62.21)
Non-Hispanic Black		1.12 (0.41–3.09)		0.54 (0.16–1.86)
Hispanic		1.95 (0.42–9.02)		0.73 (0.24–2.21)
Number of hours worked × Race/ethnicity				
Non-Hispanic White		1.00		1.00
Non-Hispanic Asian		1.00 (0.99–1.01)		0.98 (0.96–0.99)
Non-Hispanic Black		0.98 (0.96–1.00)		0.99 (0.98–1.00)
Hispanic		1.00 (0.98–1.02)		0.99 (0.98–1.00)
Multiple jobholder × Number of hours worked × Race/ethnicity				
Non-Hispanic White		1.00		1.00
Non-Hispanic Asian		0.97 (0.91–1.02)		0.96 (0.91–1.01)
Non-Hispanic Black		1.00 (0.98–1.02)		1.02 (1.01–1.05)
Hispanic		0.98 (0.95–1.01)		1.01 (0.99–1.03)

Notes: RR = relative risk. Variables included in interactions are indicated by ×. Models adjusted for age, marital status, household income, educational attainment, annual blood pressure check, body mass index, current smoking status, current drinking status, and physical inactivity.

**Fig 1:**
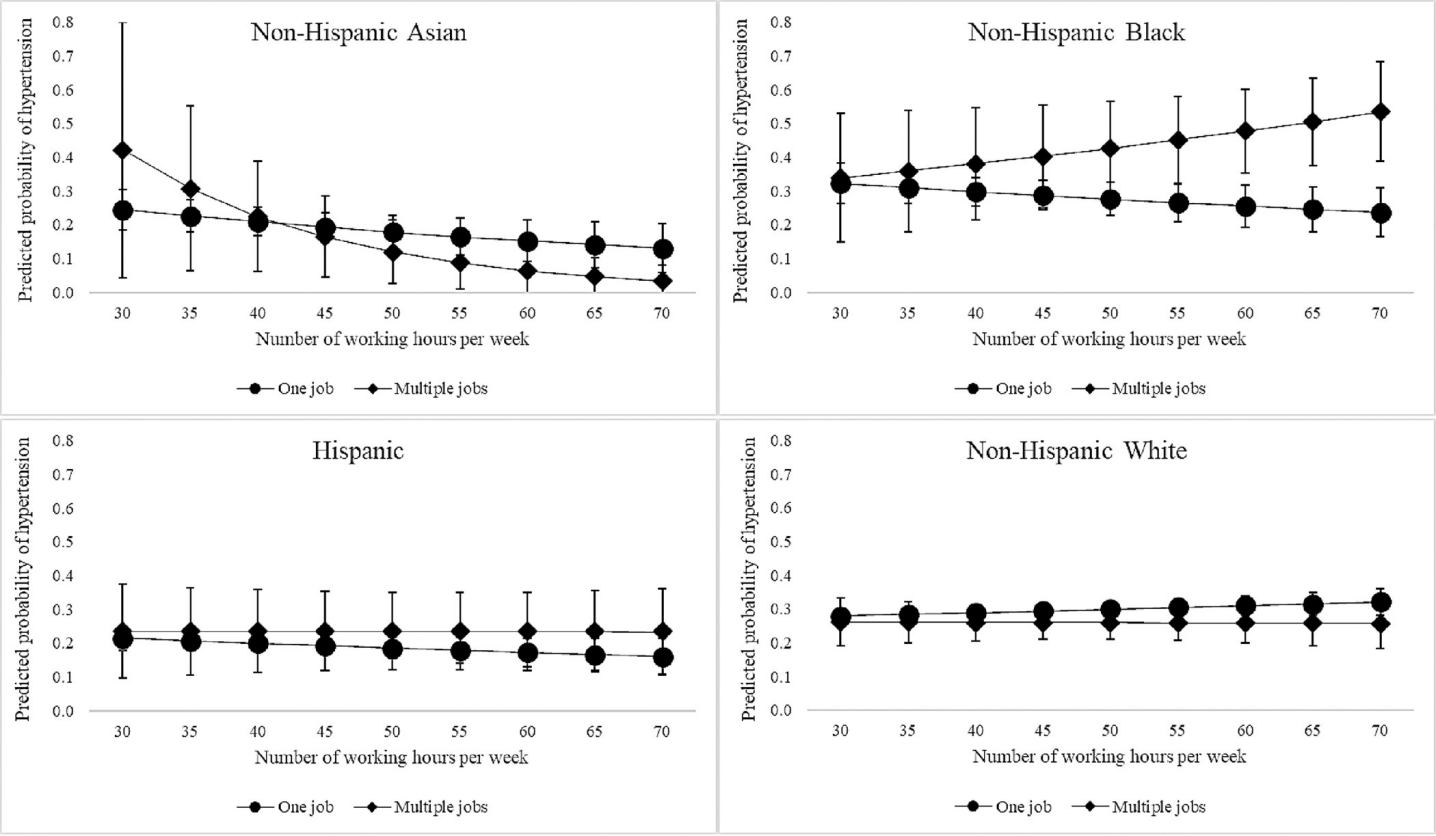
Associations between multiple jobholding and hypertension by working hours and race/ethnicity among men, National Health Interview Survey 2015. Models adjusted for age, marital status, household income, educational attainment, annual blood pressure check, body mass index, current smoking status, current drinking status, and physical inactivity.

The associations between multiple jobholding and hypertension by working hours are shown among men in Fig 1. Non-Hispanic Black men who were multiple jobholders had predicted probabilities of hypertension that were not significantly different from those who only worked one job if they worked 50 hours per week or less. However, among those who worked 60 hours per week or more, non-Hispanic Black men who were multiple jobholders had significantly higher predicted probabilities of hypertension compared to those who only worked one job. Among Non-Hispanic Asian, Hispanic, and non-Hispanic White men, there were no differences in predicted probabilities of hypertension between multiple jobholders and those who had one job at any number of weekly working hours. However, one might note that the predicted probabilities of hypertension trend down as working hours increases among multiple jobholding non-Hispanic Asian men.

## Discussion

The goal of this study was to determine if multiple jobholding was associated with higher relative risk of hypertension and to assess whether number of working hours per week was a moderator. The results showed that multiple jobholding was not associated with hypertension among women. However, there was a three-way interaction observed among men such that multiple jobholding was associated with higher predicted probabilities of hypertension among non-Hispanic Black men who had longer weekly working hours. These results suggest the need to examine the health impacts of multiple jobholding at the intersection of race/ethnicity and sex [3].

Multiple jobholding was not associated with hypertension among women. Though more women are multiple jobholders than men [2], it is possible that stress and role strain that characterize multiple jobholding in theory are not experienced by women who are multiple jobholders. It is also possible that stress and role strain are experienced by multiple jobholding women, but it does not affect self-reported hypertension among women. However, it is likely that multiple jobholding impacts other health outcomes among women [3,7–16].

Multiple jobholding was not directly associated with relative risk of hypertension among men. However, the association between multiple jobholding and hypertension was only observed among non-Hispanic Black men who worked relatively long hours. It should be noted that, in Fig 1, the difference in predicted probabilities in hypertension among non-Hispanic Black men who worked more than 50 hours per week due to an upward trend in hypertension among multiple jobholders and a downward trend among those with one job. That is, as the number of weekly working hours increased, the predicted probabilities of hypertension among non-Hispanic Black men with one job trended downward. Though this may seem unexpected, it is possible that increased working hours among non-Hispanic Black men who hold one job could align with some factor that decreases predicted probabilities of hypertension. For example, working longer hours may reflect job satisfaction or positive attitudes toward work that align with lower probabilities of hypertension. Further, longer hours may also be related to increased social support, sense of purpose, and financial compensation. It is also possible that masculinities and self-perceptions around work may be associated with longer working hours and thus result in lower probabilities of hypertension among non-Hispanic Black men who work one job for longer hours per week. This aligns with calls for more research on protective factors related to Black masculinities [40]. Moreover, it is possible that working more than 50 hours has different implications for multiple jobholders relative to those with one job. Working multiple jobs may lead to role overload or strain and increased stress as individuals try to manage multiple employment roles as well as other social roles.

This study is strengthened by the use of nationally representative data. However, the data used for this study is from 2015 and results may be different in more recent years. This may be particularly salient for adults who had multiple jobs during the COVID-19 pandemic [41]. Information on the industry of occupation, work schedules, job satisfaction, role strain, or motivations for jobholding were included. The question in NHIS used to assess multiple jobholding is potentially limited. The question simply asks, “Do you have more than one job?” Other forms of work that may not be considered a formal job are likely not considered when answering this question. For example, it is possible that having a job as well as performing informal care work may be associated with hypertension [42]. However, the study was unable to assess this.

The increase in multiple jobholding in the U.S. [1,2] warrants analyses of the associations between having more than one job and hypertension. Though multiple jobholding was not directly associated with hypertension, non-Hispanic Black men who work more than 50 hours per week and have multiple jobs have higher predicted probabilities of hypertension than those with only one job. The results of this study add to the literature on the role of work in hypertension and the impact of multiple jobholding on health in the U.S. Study findings also have policy and public health program implications. For instance, findings suggest the importance of understanding work-related social determinants of health to support programming and resources targeting multiple job holders. Future studies should examine characteristics of the work for multiple jobholders beyond number of working hours and further explore sex and gender differences in the relationship between multiple jobholding and health.
